# Traits controlling shade tolerance in tropical montane trees

**DOI:** 10.1093/treephys/tpz119

**Published:** 2019-12-19

**Authors:** Elisée Bahati Ntawuhiganayo, Félicien K Uwizeye, Etienne Zibera, Mirindi E Dusenge, Camille Ziegler, Bonaventure Ntirugulirwa, Donat Nsabimana, Göran Wallin, Johan Uddling

**Affiliations:** 1 Department of Biology, University of Rwanda, University Avenue, PO Box 117, Huye, Rwanda; 2 World Agroforestry (ICRAF), University Avenue PO Box 227, Huye, Rwanda; 3 BirdLife International, KG 501 St, PO Box 2527, Kigali, Rwanda; 4 Department of Biology, University of Western Ontario, 1157 Richmond street, London, Ontario N6A 5B7, Canada; 5 Department of Biological and Environmental Sciences, University of Gothenburg, PO Box 461, SE-405 30 Gothenburg, Sweden; 6 UMR EcoFoG, INRA, CNRS, Cirad, AgroParisTech, Université des Antilles, Université de Guyane, BP 709, 97387 Kourou Cedex, France; 7 Université de Lorraine, AgroParisTech, INRA, UMR Silva, 54000 Nancy, France; 8 Rwanda Agriculture and Animal Resources Development, PO Box 5016, Kigali, Rwanda

**Keywords:** biomass allocation, leaf temperature, plant traits, Rwanda, shade intolerance, shade tolerance, tropical montane forest

## Abstract

Tropical canopies are complex, with multiple canopy layers and pronounced gap dynamics contributing to their high species diversity and productivity. An important reason for this complexity is the large variation in shade tolerance among different tree species. At present, we lack a clear understanding of which plant traits control this variation, e.g., regarding the relative contributions of whole-plant versus leaf traits or structural versus physiological traits. We investigated a broad range of traits in six tropical montane rainforest tree species with different degrees of shade tolerance, grown under three different radiation regimes (under the open sky or beneath sparse or dense canopies). The two distinct shade-tolerant species had higher fractional biomass in leaves and branches while shade-intolerant species invested more into stems, and these differences were greater under low radiation. Leaf respiration and photosynthetic light compensation point did not vary with species shade tolerance, regardless of radiation regime. Leaf temperatures in open plots were markedly higher in shade-tolerant species due to their low transpiration rates and large leaf sizes. Our results suggest that interspecific variation in shade tolerance of tropical montane trees is controlled by species differences in whole-plant biomass allocation strategy rather than by difference in physiological leaf traits determining leaf carbon balance at low radiation.

## Introduction

Tropical forests host a high diversity of species and are extremely productive ecosystems, accounting for about one-third of global terrestrial gross primary production (Dirzo and Raven 2003, Lewis 2006, Beer et al. 2010, Malhi et al. 2013). One reason for the high diversity and productivity of tropical forests is that they harbor tree species with strongly varying degrees of shade tolerance, forming complex canopies with multiple layers and pronounced gap dynamics (Osunkoya et al. 1994, Gommers et al. 2013, Valladares et al. 2016). Shade tolerance has been attributed to many different plant traits that maximize carbon gain and/or increase stress tolerance and survival rates under low light. At present, however, we lack a clear understanding of which traits are most important in controlling interspecific variation in shade tolerance (Valladares and Niinemets 2008, Valladares et al. 2016). Previous studies have often focused on either leaf physiological traits or whole-plant biomass allocation and structure (Valladares and Niinemets 2008). Furthermore, it remains unclear if there is a difference in light acclimation capacity between shade-tolerant (ST) and shade-intolerant (SI) tropical tree species, with conflicting reports in the literature (Rozendaal et al. 2006, Coste et al. 2009, 2010, Houter and Pons 2014, Dusenge et al. 2015). This is partly because, in many previous studies, ST species were not grown at high light and SI species not at low light levels (Poorter et al. 2019). Moreover, traits change with age and size (Houter and Pons 2012), complicating the use of sun- and shade-leaf data from mature trees for predicting plasticity and shade-tolerance of juvenile trees. There is thus a need for new experimental studies exploring the contributions of different plant traits (i.e., leaf vs whole-plant level, and physiological vs structural) in controlling shade tolerance of tropical trees (Valladares and Niinemets 2008, Valladares et al. 2016, Poorter et al. 2019).

Two main hypotheses on the suites of traits responsible for species shade tolerance have been proposed: the carbon gain hypothesis and the stress tolerance hypothesis (Valladares and Niinemets 2008). The carbon gain hypothesis states that shade tolerance is the consequence of high light-use efficiency resulting from traits maximizing carbon gain and minimizing carbon losses in a low-light environment. Such traits include high quantum yield of photosynthesis (QY) and low leaf respiration leading to low photosynthetic light compensation point (LCP), low leaf mass per unit area (LMA) and a high fraction of biomass invested in organs contributing to light interception in the understorey (i.e., leaves and branches; Givnish 1988, Kitajima 1994, Lusk and Pozo 2002, Niinemets 2006, Baltzer and Thomas 2007*b*, Luttge 2008, Valladares and Niinemets 2008). The stress tolerance hypothesis attributes shade tolerance to high investments in traits that maximize the resistance to biotic and abiotic stresses in the understorey (Kitajima 1994; Gommers et al. 2013). Such traits include high concentrations of defense metabolites and high wood density and LMA. The two hypotheses are not necessarily mutually exclusive and it is plausible that both contribute to explain shade tolerance.

The carbon gain hypothesis has been very influential for our understanding of shade tolerance, but is currently under challenge. It originally predicts that ST species should have higher relative growth rates (RGR) than SI species when growing in the shade (Givnish 1988), but in most studies lower RGR was observed in ST seedlings when growing in both high and low light conditions (Kitajima 1994, Walters and Reich 1999, Kunstler et al. 2016). The carbon gain hypothesis also suggests lower LMA for ST compared with SI species to maximize light interception (Givnish 1988, Valladares and Niinemets 2008, Poorter et al. 2009), but some studies with tropical trees found the opposite (Coste et al. 2005, Kitajima 1994, Mao et al. 2014). This suggests that the conservation of carbon is more important than the efficiency of its capture (Lusk et al. 2011, Reich et al. 2003), which is more in line with the stress tolerance hypothesis. Some studies have indeed reported that plant survival in the understorey is poorly linked to whole-plant carbon gain (Kitajima 1994, Reich et al. 2003). In addition, contrary to the carbon gain hypothesis but in agreement with the stress tolerance hypothesis, some studies have indicated that ST plants invest relatively more biomass into roots than SI species when growing in the understorey (Poorter et al. 2010, Mao et al. 2014).

Leaf physiological data also sometimes conflict with the carbon gain hypothesis. The hypothesis predicts higher QY and lower respiration and LCP for ST compared with SI species (Valladares and Niinemets 2008), but Walters and Reich (1999) could not find any significant differences in these traits between the two groups. Moreover, in a study on tropical montane rainforest tree species differing in shade tolerance, higher chlorophyll content per unit leaf area in ST compared with SI species did not translate into higher apparent QY (i.e., the initial slope of the light response of net photosynthesis; Dusenge et al. 2015). This suggests that the common observation of higher chlorophyll content in ST species (Niinemets 1997, Valladares and Niinemets 2008) does not necessarily contribute to increased leaf carbon gain. All these findings show that new and more holistic studies—including side-by-side comparisons of different types of traits across species with a broad range of shade tolerance—are needed to explore which traits and strategies are most important in controlling tropical tree shade tolerance (Valladares et al. 2016).

Tropical rainforest tree species—adapted to temporally stable climatic conditions—have been suggested to have a more narrow optimum temperature range compared with tree species from more seasonal climates (Doughty and Goulden 2008, Wright et al. 2009). It has further been proposed that they operate near their thermal optimum, above which their CO_2_ assimilation and growth may decline (Clark et al. 2010, Way and Oren 2010). Indeed, photosynthesis was negatively affected by warming in both branch chamber experiments (Doughty 2011) and indoor growth chamber experiments (Cheesman and Winter 2013*a*, Slot and Winter 2017). Moreover, daytime leaf temperatures greatly exceeded both air temperature and the optimal temperature for photosynthesis in a common garden experiment with tropical tree seedlings (Vårhammar et al. 2015). This exceedance was particularly high in sun-exposed ST species with low transpiration rates and large leaf size. Controlled experiments also indicate that tropical ST species are more sensitive to warming than SI species (Doughty 2011, Cheesman and Winter 2013*b*). It is therefore possible, but hitherto unexplored, that high leaf temperatures result in physiological heat stress and lower competitiveness of ST species growing in high radiation environments.

The overall aim of this study was to explore which traits were most important in controlling species shade tolerance in tropical montane trees. The specific hypotheses were as follows: (i) ST species have a whole-plant biomass allocation strategy that maximizes light interception when grown in the understorey, i.e., higher relative investment into branches and leaves. (ii) ST species have physiological leaf traits that allow for a more favorable leaf carbon balance in a low-light environment, i.e., lower respiration and LCP. In addition, a third hypothesis related to high radiation intolerance was included to explore if ST species might suffer under open sky conditions: (iii) ST species have leaf traits that cause high leaf temperature (i.e., low transpiration rates and large leaf size) and consequent physiological heat stress under high sun exposure. To address these hypotheses, we investigated a broad range of traits in seedlings of six tropical montane tree species with varying degrees of shade tolerance grown under three different radiation regimes: open sky, and beneath either sparse or dense overstorey canopies. Traits measured include structural, chemical and physiological leaf traits as well as whole-plant growth and biomass allocation.

## Materials and methods

### Study site and environmental measurements

The experiment was conducted in the Arboretum of Ruhande (‘Arboretum’ in the following), Rwanda (altitude: 1638–1737 m; latitude 2°36′S and longitude 29°44′E). The Arboretum was established in 1933 after the relocation of the population that lived and farmed in that area (Nsabimana 2009). The plantation includes 216 tree species (169 exotic and 47 native) and covers an area of 200 ha that is subdivided into around 500 plots of 50 × 50 m, most of which are monospecific (Nsabimana et al. 2008, Dusenge et al. 2015). The plants of the present study originate from seeds collected in Nyungwe National Park (‘Nyungwe’ in the following), a tropical montane rainforest that covers an area of approximately 1000 km^2^ and is located in southwestern Rwanda 50–100 km west of the Arboretum (2°17′−2°50′S, 29°07′−29°26′E, 1600–2950 m altitude). The Arboretum is within the altitudinal range of all six species studied (see below).

The long-term climate at the Arboretum is characterized by small variations in monthly mean air temperature and a bimodal rain pattern, with one strong dry period from mid-June to mid-August and a less distinct dry period in January and February (Nsabimana 2009). Mean daytime and 24-h air temperatures during 2013–17 were 21.1 and 19.5 °C, respectively. The difference in mean temperature between the warmest and coldest months was 1.5 °C. Mean daytime vapor pressure deficit of the air (VPD) was 1.02 kPa, mean annual rainfall 979 mm and mean daytime photosynthetic photon flux density (PPFD) 733 μmol m^−2^ s^−1^ during the same period. At a meteorological station located at 2465 m altitude in Nyungwe, mean daytime and 24-h air temperatures were 15.6 and 14.3 °C, respectively, mean daytime VPD 0.39 kPa, mean annual rainfall 1879 mm and mean daytime PPFD 633 μmol m^−2^ s^−1^ during 2007–13. More information on meteorological conditions at both Arboretum and Nyungwe was provided by Mujawamariya et al. (2018).

In addition, a weather station (VP-3, PYR and ECRN-100, Decagon Device, Inc., Pullman, WA, USA) recorded air temperature, relative humidity, solar radiation and precipitation at another open place by the Arboretum office during the last quarter of the study. These data were used for comparisons with leaf temperature measurements (see below).

Seven Tiny Tags (Model Tinytag Plus 2, Gemini Data Loggers Ltd, Chichester, UK) recorded air temperature and relative humidity at 1.8 m in plots with different radiation regimes (six plots with different overstorey canopy transmission plus one open plot; see below) during 15 successive days in March 2016. To estimate relative radiation transmission of the overstorey in each plot, below-canopy radiation at the position of each seedling was measured with a portable quantum meter (MQ-500, Apogee Instruments, Inc., Logan, Utah, USA) during 12:00–14:00 h on two sunny days, one in the wet and one in the dry season.

### Plant materials and experimental design

The six montane tree species used in this species represent common ST and SI species in Nyungwe, as judged from their abundance in forest stands at different successional stages in the forest (Nyirambangutse et al. 2017 and references therein). The ST species are all among the 20 most common species in Nyungwe, together comprising 25% of the large trees (diameter at breast height >30 cm) in the forest. The ST species, which are more abundant in late-successional stands, were *Carapa grandiflora* (*Cg*; family: Meliaceae; altitudinal range in Rwanda: 1600–2500 m), *Entandrophragma excelsum* (*Ee*; family: Meliaceae; altitudinal range in Rwanda: 1500–2100 m) and *Syzygium guineense* spp. *parvifolium* (*Sg*; family: Myrtaceae; altitudinal range in Rwanda: 1000–2100 m). The SI species, which are more abundant in early-successional stands, were *Croton megalocarpus* (*Cm*; family: Euphorbiaceae; altitudinal range in Rwanda: 1600–2400 m), *Dombeya goetzenii* (*Dg*; family: Malvaceae; altitudinal range in Rwanda: 1300–3000 m) and *Polyscias fulva* (*Pf*; family: Araliaceae; altitudinal range in Rwanda: 1200–2400 m). Species’ altitudinal range were taken from Bloesch et al. (2009) or Fischer and Killmann (2008). Since shade tolerance varies along a continuum, classification of species into distinct successional groups is not straightforward. While *Cm* and *Dg* are typical short-lived early-successional species, trees of *Pf* can grow quite large and may stay around longer after canopy closure. Although all three ST species are more abundant in late-successional stands, *Sg* is also quite common in early-successional stand. We therefore did not apply successional group identity (ST versus SI) in the statistical analysis (see Statistical analyses section below).

The plants, grown from seeds collected in Nyungwe, were first cultivated for 3.5 months at the Arboretum’s nursery, in small pots that were irrigated daily and contained < 1 l of clayey soil from the surrounding and added organic fertilizer. In March–April 2015, they were transplanted into 10-l pots with local clayey soil. Plants were randomly distributed to nine different plots in the Arboretum, differing in overstorey leaf area index and canopy light transmittance (Table 1). These nine plots were divided into three radiation regimes: three open plots, three plots with the overstorey consisting of rather sparse canopies of early-successional species (*Dg*, *Cm* and *Prunus africana*), and three plots with the overstorey consisting of dense canopies of later-successional species (*Cg*, *Sg* and *Magnistipula butayei*). Each species had six replicate seedlings in each plot and the total number of seedlings in the experiment was thus 324 (3 radiation regimes × 3 plots in each regime × 6 species × 6 replicates).

**Table 1:** Comparison of environmental conditions of the nine plots of the study. Temperature values(*T*_air_) are means over 15 days with simultaneous measurements in all plots. Data on PPFD were recorded between 12:00 and 14:00 h on two sunny days, one in August (dry season) and one in January (wet season).

Treatment	Plot^a^	*T*_air_ day (°C)	*T*_air_ night (°C)	PPFD, dry season, mid-day (μmol m^−2^ s^−1^)	PPFD, wet season, mid-day (μmol m^−2^ s^−1^)	Light transmission (%), on dry/wet season days
Open field	Open 1	24.2^b^	16.1^b^	1970	2059	100/98
	Open 2	24.2^b^	16.1^b^	1806	2044	95/97
	Open 3	24.2^b^	16.1^b^	1990	1979	98/95
Sparse canopy	*Cm*	21.2	18.0	853	789	42/38
	*Dg*	21.3	15.5	670	138	31/7
	*Pa*	22.1	15.2	932	409	43/19
Dense canopy	*Cg*	21.6	17.8	258	179	13/9
	*Mb*	21.6	17.7	216	278	11/13
	*Sg*	21.4	16.6	233	117	11/6

Light transmission is the plot radiation divided by the open sky radiation at a large open area 50 m outside the Arboretum of Ruhande.

^a^Open 1, 2 and 3 are the open plots; *Cm*, *Dg* and *Pa* are the sparse canopy plots with overstorey consisting of *Croton megalocarpus*, *Dombeya goetzenii* and *Prunus africana*; *Cg*, *Mb* and *Sg* are the dense canopy plots with overstorey consisting of *Carapa grandiflora*, *Magnistipula butayei* and *Syzygium guineense*.

^b^Data from the same weather sensor.

Pots were placed 0.7 m apart to minimize shading among seedlings. Plants were regularly irrigated to assure high soil water availability throughout the study. During the 3-month dry season, seedlings in open plots were watered daily while those in sparse and dense canopy plots were watered three times per week. During the rest of the year, plants were watered regularly and as needed depending on rainfall. No fertilizer was added during the shade tolerance experiment. This may have caused increased nutrient constraints in fast-growing plants in open and sparse canopy plots. However, such constraints were probably not unrealistically large since our findings of decreased mass-based but increased area-based leaf nitrogen (N) content of plants in high radiation regimes (see below) is the typical observation also in freely rooted plants (Poorter et al. 2019).

Monthly health inspections were conducted from May 2015 to April 2016. In total, 21 out of the 324 seedlings died during the experiment. All plants were harvested in May 2016.

### Gas exchange measurements and calculations

Leaf gas exchange was measured in three plants per species in each plot during February–April 2016, using a portable LI-6400 leaf gas exchange instrument (LI-COR, Lincoln, NE, USA). Two leaves per plant were measured: one for net photosynthesis (*A*_n_) and one for dark respiration (*R*_d_). The selected leaves were at least 2 months old, fully developed and had comparatively healthy appearance. The *A*_n_ measurements to determine photosynthetic capacity (i.e., *V*_cmax_: maximum rate of Rubisco carboxylation, and *J*_max_: maximum rate of photosynthetic electron transport) were done at a PPFD of 1800 μmol m^−2^ s^−1^, an air flow of 400 μmol s^−1^ and the block temperature of the instrument at 25 °C. In two out of three plants per species and plot, measurements were done at three different CO_2_ concentrations of the air entering the leaf chamber: 410, 200 and 2000 μmol mol^−1^. For the third plant, photosynthesis was only measured at 410 μmol mol^−1^. Measurements were conducted within a few minutes after clamping onto the leaf or changing CO_2_ concentration, without waiting for stomatal conductance to respond to respond to altered environmental conditions. Data of *A*_n_ in shaded plots (and the derived *V*_cmax_ and *J*_max_ values) are thus representative for photosynthesis during sunflecks and do not reflect values where both photosynthesis and stomata have fully responded to the high measurement radiation. The stomatal conductance data point reported is that measured at 410 μmol mol^−1^ CO_2_ concentration. The photosynthesis model by Farquhar et al. (1980), with modifications of photosynthetic temperature dependencies by Bernacchi et al. (2001), was parameterized using the one-point method (De Kauwe et al. 2016). The measurements at 200 and 2000 μmol mol^−1^ CO_2_ concentrations were used to determine *V*_cmax_ and *J*_max_, respectively, and tree-specific *R*_d_ data described below (i.e., dark respiration) were used in both cases. The measurements at the 410 μmol mol^−1^ CO_2_ concentration were used only for determination of light-saturated *A*_n_, not for *V*_cmax_ or *J*_max_.

On the same leaf measured for *A*_n_ at different CO_2_ concentrations, the initial light response of *A*_n_ was also measured at PPFD of 25 and 75 μmol m^−2^ s^−1^, the CO_2_ concentration at 410 μmol mol^−1^ and the block temperature of the instrument at 25 °C. These data were originally planned to be used for the determination of apparent QY and LCP. However, subsequent tests with measurements at 25, 50 and 75 μmol m^−2^ s^−1^ showed that the light response was not linear in the entire PPFD range of 25–75 μmol m^−2^ s^−1^. We therefore do not report any QY results, while LCP was instead calculated by linear intrapolation between the *A*_n_ measurement at a PPFD of 25 μmol m^−2^ s^−1^ and *R*_d_ measured on a neighboring leaf on the same plant (see below). The method does not account for the Kok effect (Kok 1948), which is difficult to determine for large numbers of plants in the field since it requires several data points around the LCP. Although not ideal, this use of two data points from two neighboring leaves to derive LCP is not likely to cause significant systematic bias with respect to species or radiation treatments.

Before *R*_d_ measurements, leaves were dark acclimated by covering them with tinfoil for at least 30 min. This measurement was done with an air flow of 250 μmol s^−1^, CO_2_ concentration of 410 μmol m^−2^ s^−1^ and leaf chamber block temperature of 25 °C. After the measurement of each plant, an empty chamber measurement was done to allow for corrections of possible leaks and gasket diffusion.

### Other leaf trait measurements

Leaf temperature was measured on all plants in all plots at 12:00–15:00 h on three sunny days, using three infrared thermometers (Model: BP 10, Trotec, Heinsberg, Germany). Three people measured simultaneously, one in each radiation regime (open sky, sparse canopy and dense canopy) and then rotating such that each person measured one plot of each regime. The infrared thermometer had a 6:1 ratio of the distance to the surface compared with the diameter of the surface area being measured. It was held about 6 cm from the leaf surface and pointed towards the leaf without shading it, aiming at the central position on one of the leaf halves for large leaves and at the middle for small leaves. The leaf was horizontally positioned (= 0°) and the angle of the infrared thermometer was held at 45° relative to the leaf.

On all leaves measured for gas exchange, leaf discs were sampled (three discs with 18 mm diameter for large leaves or five discs with 10 mm diameter for smaller leaves) for LMA determination and subsequent nutrient and stable isotope analyses. Leaf samples were oven-dried at 70 °C for at least 72 h. After determining LMA, leaf discs and remaining leaf material was ground into a fine powder with a ball mill grinder (MM 301, MM 200, Retsch, Germany). In total, 160 samples were sent for analyses of leaf N and stable carbon isotope composition using a continuous flow isotope ratio mass spectrometer (UC Davis Stable Isotope Facility, Davis, CA, USA). Carbon isotopes were used to estimate plant intrinsic WUE (iWUE). Analysis of 37 essential non-N elements was done for one combined sample per species (except *Dg* where there was too little leaf sample material) and plot, using inductively coupled plasma mass spectrometry (ACME Analytical Laboratories, Vancouver, Canada). Out of these non-N elements, only data for phosphorus are reported. The iWUE determined based on carbon isotope data was calculated as the difference between the ambient CO_2_ concentration and intracellular CO_2_ concentration times 1.6 to account for the difference in stomatal conductance of water vapor compared with CO_2_. Ambient CO_2_ was assumed to be 402 μmol mol^−1^, and intercellular CO_2_ was calculated using equations in Farquhar et al. (1989).

### Biomass harvest

In the harvest at the end of experiment, we first measured stem diameter and height and counted the number of attached leaves in each living plant. Leaf angles of five leaves per plant at randomly selected canopy positions were also measured and leaf discs were taken from the same leaves for LMA determination and chemical analyses. A SPAD-502 meter (Konica Minolta Sensing, Inc., Ltd, Osaka, Japan) was used to measure the SPAD value (a proxy of area-based leaf chlorophyll content) of one fully developed, healthy and at least 2-month-old leaf per plant. Stem base diameter and height were measured using a ruler and digital caliper, respectively. The seedlings were then harvested and divided into roots, main stem, branches, petioles and leaves. Plant parts were oven-dried at 70 °C until constant mass. Wood density of dry wood was determined based on measurements of mass and volume on a piece of a dry stem without bark from each plant.

In addition, eight plants per species which were not included in the main experiment were harvested in April 2015 to determine the initial biomass of different plant parts at the beginning of the experiment. The mean total initial dry mass ranged from 5 g to 14 g in the six species (Table S1 available as Supplementary Data at *Tree Physiology* Online).

### Statistical analyses

Data were statistically analyzed using analysis of variance (ANOVA) with radiation regime as a fixed factor and plot and species as random factors. The linear model of the experiment was the following:

*y* = *μ* + *R*_i_ + *S*_j_ + *P*(*R*)_k(i)_ + *RS*_ij_ + *SP*(*R*)_jk(i)_ + *e*_l(ijk)_,

where *y* is the response variable, *μ* is the intercept, *R* is radiation regime, *S* is species identity, *P* is plot, *e* is individual random variation and the subscripts indicate the levels of each factor. Plots were nested within radiation regimes and plant replication for each species in a plot was six. With the experimental design used, *F* ratios for the effects of *S*_j_, *P*(*R*)_k(i)_ and *RS*_ij_ were calculated using *SP*(*R*)_jk(i)_ as the error term, while the *F* ratio for *SP*(*R*)_jk(i)_ was calculated using *e*_l(ijk)_ as the error term (Underwood 1997). Significant species by radiation interactions were found for most variables, and main effects of radiation were tested by simple one-way ANOVA within each species individually. Effects were considered statistically significant at *P* ≤ 0.05.

## Results

### Environmental conditions

Daytime temperatures at 1.8 m above ground were 2–3 °C lower in both sparse and dense canopy plots compared with open plots (Table 1). Nighttime temperatures were on average 1.1–1.3 °C higher in dense canopy plots compared with open or sparse canopy plots. Nighttime temperature was lowest in two of the sparse canopy plots as a result of their lower position in the Arboretum area, just at the end of a downhill slope. On average, across two measurement days, dense canopy plots had 10% midday canopy transmission while sparse canopy plots had 30%. Daytime measurements made during the dry season in August showed marked differences in canopy transmission between all sparse versus dense canopy plots. In the wetter season in January, however, one of the sparse canopy plots (with *Dg* as overstorey species) had canopy transmission comparable to that in dense canopy plots. This difference between August and January was a result of high seasonality in both leaf shedding and leaf production in *Dg*. On average across the two measurement days, however, the *Dg* sparse canopy plot had higher canopy transmission than those in all three dense canopy plots.

### Biomass, allocation and health inspections

**Figure 1.**
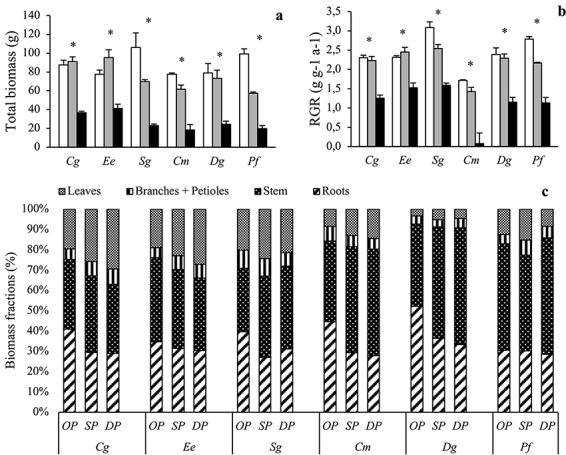
(a) Total biomass (g), (b) relative growth rate (RGR) and (c) biomass allocation (%) of *Carapa grandiflora* (*Cg*), *Entandrophragma excelsum* (*Ee*), *Syzygium guineense* (*Sg*), *Croton megalocarpus* (*Cm*), *Dombeya goetzenii* (*Dg*) and *Polyscias fulva* (*Pf*) planted in open (white in a, b; OP in c), sparse canopy (gray in a, b; SP in c) and dense canopy (black in a, b; DP in c) plots. Species to the left (*Cg*, *Ee*, *Sg*) are shade tolerant and species to the right (*Cm*, *Dg*, *Pf*) shade-intolerant. Leaf biomass data represent leaves attached at the time of harvest. The error bars represent standard errors (*n* = 3). The symbol * in (a) and (b) indicates significant variation among radiation regimes within a species. Overall statistics results are provided in Table 2.

All tree biomass and structure parameters exhibited significant (i.e., *P* ≤ 0.05) or, in two cases, near-significant (0.050 < *P* ≤ 0.068) species by radiation interactions (Table 2), meaning that effects of shade were species-dependent. The radiation regime had a significant influence on total tree biomass at harvest (Figure 1a) and RGR (Figure 1b) in all species. However, the reduction in RGR in dense canopy compared with open plots was smallest in the ST species *Ee* (34%) and greatest in the SI species *Cm* (95%). In the other four species, the RGR reductions were intermediate but somewhat larger in the SI species *Pf* (59%) and *Dg* (52%) than in the ST species *Sg* (49%) and *Cg* (46%).

**Table 2:** *P* values for effects on different plant traits according to the ANOVA. Brackets indicate that the preceding factor is nested inside the factor within brackets; × indicate interacting effects of the preceding and following factors.

Plant trait	*P* values for different sources of variation			
	Species	Plot (radiation)	Species × radiation	Species × plot (radiation)
				
Tree size and structure				
Total	**0.001**	0.199	**0.008**	**0.0001**
Leaves	**<0.0001**	**0.008**	**0.002**	**0.001**
Branches + petioles	**<0.001**	0.232	**0.002**	0.253
Stem	**0.268**	0.489	**0.006**	**0.001**
Roots	**0.023**	**0.005**	**0.015**	**<0.0001**
RGR	**<0.0001**	0.183	0.052	**<0.0001**
% leaves	**<0.0001**	**0.004**	0.068	**<0.0001**
% branches + petioles	0.076	**0.014**	**0.029**	0.127
% stem	**<0.0001**	0.355	**0.003**	**<0.0001**
% roots	**0.002**	**0.0001**	**0.007**	**0.029**
Root:shoot ratio	**0.002**	**0.001**	**0.012**	**0.014**
Stem diameter	**<0.0001**	0.158	**0.030**	**0.014**
Stem height	**<0.0001**	0.087	**0.020**	**0.0002**
Stem height:diameter ratio	**<0.0001**	0.084	**0.048**	**<0.0001**
Height increment	**<0.0001**	**0.036**	**0.001**	**0.001**
Canopy leaf number	**<0.001**	**0.006**	**0.0001**	**0.002**
Wood density	**<0.0001**	0.251	**0.003**	0.351
				
Leaf structure				
LMA	**<0.001**	**<0.001**	**<0.001**	**0.007**
Leaf mass	**<0.001**	0.140	**0.0004**	0.174
Leaf area	**<0.001**	**0.0004**	**0.0002**	**0.006**
Leaf length	**<0.001**	**0.032**	0.176	0.082
Leaf width	**<0.001**	0.317	**0.003**	0.447
Leaf thickness	**<0.001**	**<0.001**	0.503	0.599
Leaf angle	**<0.001**	0.063	**0.002**	**0.050**
				
Leaf physiology				
*A*_n_, light-saturated	**<0.001**	**<0.001**	**0.002**	0.872
*V*_cmax_	**0.006**	0.086	**0.006**	0.720
*J*_max_	0.198	0.616	0.075	**0.037**
*J*_max_:*V*_cmax_ ratio	**0.004**	**0.010**	0.262	0.165
*R*_d_, area-based	0.400	**0.003**	0.795	0.115
*R*_d_, mass-based	<0.001	**0.006**	0.750	0.142
LCP	0.593	**0.041**	0.704	0.374
*g*_s_	**<0.001**	**<0.001**	0.115	0.490
iWUE	**<0.001**	**<0.001**	**0.015**	0.082
Leaf chemistry				
N_a_	**<0.001**	0.784	0.332	**0.022**
N_m_	**<0.001**	0.090	0.551	**0.005**
P_a_	**0.0002**	0.163	0.070	**<0.001**
P_m_	**0.0003**	0.101	**0.040**	**<0.001**
SPAD	**<0.001**	**0.002**	**0.005**	0.105

Figures in bold indicate significant *P* values.

*V*_cmax_: maximum rates of photosynthetic carboxylation, *J*_max_: maximum rates of electron transport, *R*_d_: leaf dark respiration, LCP: light compensation point, *g*_s_: stomatal conductance, *A*_n_: light-saturated net CO_2_ assimilation, P_a_: leaf phosphorus content per unit area, P_m_: leaf phosphorus content per unit mass, N_a_: leaf nitrogen content per unit area, N_m_: leaf nitrogen content per unit mass, LMA: leaf mass per unit leaf area, SPAD: proxy for leaf chlorophyll content, RGR: relative growth rate.

The ST species all exhibited lower stem biomass fractions but higher fractions of leaves and branches plus petioles compared with the SI species (Figure 1c). The ST species *Cg* and *Ee* responded to shading by increasing the fractions of leaves, branches and petioles, a strategy not observed in the other species. The SI species plus *Sg* instead responded to shading by increasing their stem biomass fractions. Both ST and SI species had similar fractional investments into roots and all except *Ee* and *Pf* responded to shading by decreasing this fraction.

Monthly health inspections showed that some seedlings suffered from different kinds of health problems at some point during the experiment, including insect infection (4 plants) and herbivory (8 plants), partial dehydration (22 plants) and mammal herbivory (41 plants). Insect infection or herbivory caused no significant reduction in total biomass at harvest. Partially dehydrated plants either recovered well or subsequently died and were excluded from the biomass analysis. Seedlings affected by mammal herbivory had 21% lower total biomass at the final harvest, and these were therefore removed from the biomass analysis. A total of 21 out of 324 trees died throughout the experiment (Figure S1 Supplementary data for this article are available at *Tree Physiology* Online). Twelve of these trees were in the dense canopy plots and 14 belonged to SI species (i.e., *Cm*, *Dg*, *Pf*), but differences among species or treatments were not statistically significant.

Dry wood density was lowest in the SI species *Dg* and *Pf* and generally decreased with shading (Table S2 available as Supplementary Data at *Tree Physiology* Online).

### Leaf structural, chemical and physiological traits

**Figure 2.**
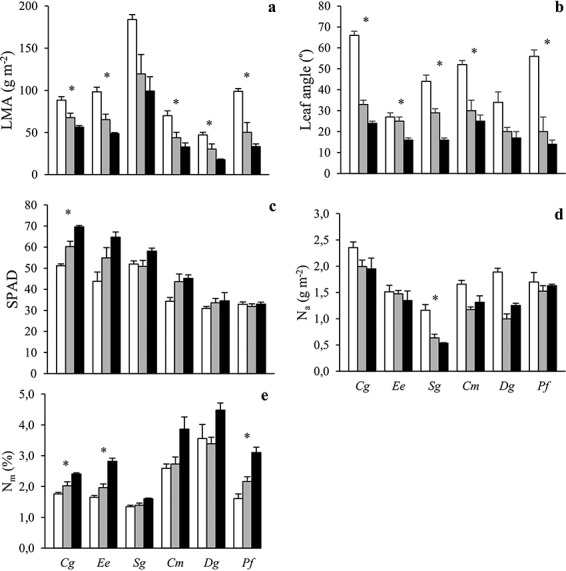
The (a) leaf mass per unit leaf area (LMA), (b) leaf angle, (c) SPAD, (d) nitrogen content per unit leaf area (N_a_) and (e) nitrogen content per unit leaf mass (N_m_) of six tropical tree species grown in open (white), sparse canopy (gray) and dense canopy (black) plots. Species to the left (*Cg*, *Ee*, *Sg*) are shade-tolerant and species to the right (*Cm*, *Dg*, *Pf*) shade-intolerant. The error bars represent standard errors (*n* = 3). The symbol * indicates significant variation among radiation regimes within a species. Species: *Carapa grandiflora* (*Cg*), *Entandrophragma excelsum* (*Ee*), *Syzygium guineense* (*Sg*), *Croton megalocarpus* (*Cm*), *Dombeya goetzenii* (*Dg*) and *Polyscias fulva* (*Pf*).

Values of LMA were highest in *Sg*, lowest in *Cm* and *Dg*, and quite similar in the remaining three species (Figure 2a). It declined with shading in all species. Mean leaf angles (horizontal = 0°) were below or about 30° in all plants growing beneath sparse or dense canopies (Figure 2b). They were considerably steeper in open plots in all species and the highest values were found for Cg (66°) followed by Pf (56°) and Cm (52°). Neither mean leaf angles nor their sun-shade plasticity differed in a systematic way between ST and SI species. SPAD values were higher in the ST compared with the SI species (Figure 2c). Moreover, there was a significant species by radiation interaction on SPAD (Table 2), with all species except the SI species *Dg* and *Pf* exhibiting increases in SPAD under low radiation conditions. The ST species thus had both intrinsically high chlorophyll content and an ability to increase these levels further upon shading.

Mass-based leaf N concentration was generally higher under shading while the opposite was sometimes true for area-based leaf N content (Figure 2d and e). There were no species by radiation interaction on area- or mass-based leaf N (Table 2). Area-based leaf N was weakly positively correlated with both photosynthetic capacity parameters: *V*_cmax_ and *J*_max_ (Figure S2 available as Supplementary Data at *Tree Physiology* Online). For leaf phosphorus (P), mass-based concentrations were highest in *Ee* trees growing in sparse canopy plots and area-based P content was generally lowest in dense canopy plots (Figure S3 available as Supplementary Data at *Tree Physiology* Online). Species by radiation interactions had *P* values of 0.040 and 0.070 for mass- and area-based leaf P, respectively (Table 2).

**Figure 3.**
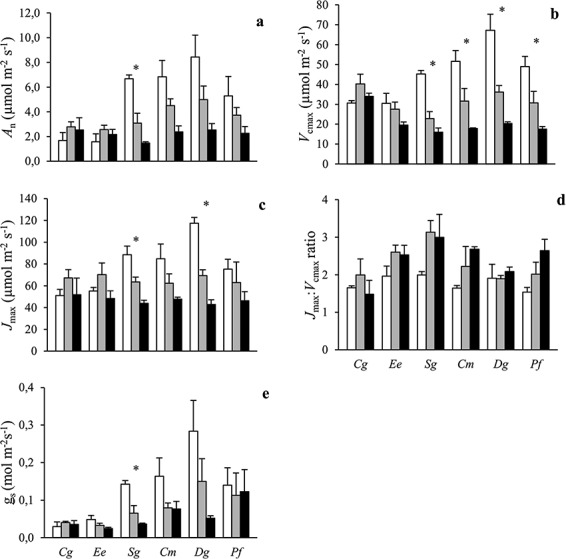
The (a) light-saturated net CO_2_ assimilation (*A*_n_), (b) maximum rates of photosynthetic carboxylation (*V*_cmax_) and (c) electron transport (*J*_max_), (d) *J*_max_:*V*_cmax_ ratio and (e) stomatal conductance (*g*_s_) of six tropical tree species grown in open (white), sparse canopy (gray) and dense canopy (black) plots. Species to the left (*Cg*, *Ee*, *Sg*) are shade tolerant and species to the right (*Cm*, *Dg*, *Pf*) shade-intolerant. The error bars represent standard errors (*n* = 3). The symbol * indicates significant variation among radiation regimes within a species. Species: *Carapa grandiflora* (*Cg*), *Entandrophragma excelsum* (*Ee*), *Syzygium guineense* (*Sg*), *Croton megalocarpus* (*Cm*), *Dombeya goetzenii* (*Dg*) and *Polyscias fulva* (*Pf*).

In open plots, light-saturated *A*_n_, *V*_cmax_ and *J*_max_ were higher in the three SI species (i.e., *Cm*, *Dg*, *Pf*) and *Sg* than in *Cg* and *Ee* (Figure 3a–c). In dense canopy plots, however, values were mostly similar among species. In the ST species *Cg* and *Ee*, photosynthetic parameters were mostly equal in the three radiation regimes while in the other species they decreased with shading. These interspecific differences in shading responses resulted in significant (*A*_n_, *V*_cmax_) or nearly significant (*J*_max_, *P* = 0.075) species by radiation interactions (Table 2). The *J*_max_:*V*_cmax_ ratios also differed among species but not in a pattern consistent with species shade tolerance (Figure 3d). It was generally higher under shading and there was no significant difference in radiation responses among species (*P* = 0.26; Table 2). Stomatal conductance exhibited similar patterns to *A*_n_ except that SI species had higher values than ST species also in the dense canopy plots (Figure 3e).

**Figure 4.**
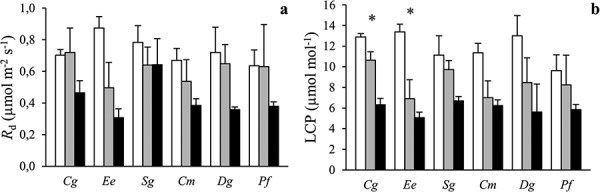
The (a) leaf dark respiration (*R*_d_) and (b) photosynthetic light compensation point (LCP) of six tropical tree species grown in open (white), sparse canopy (gray) and dense canopy (black) plots. Species to the left (*Cg*, *Ee*, *Sg*) are shade tolerant and species to the right (*Cm*, *Dg*, *Pf*) shade-intolerant. The error bars represent standard errors (*n* = 3). The symbol * indicates significant variation among radiation regimes within a species. Species: *Carapa grandiflora* (*Cg*), *Entandrophragma excelsum* (*Ee*), *Syzygium guineense* (*Sg*), *Croton megalocarpus* (*Cm*), *Dombeya goetzenii* (*Dg*) and *Polyscias fulva* (*Pf*).

Dark respiration did not vary much among species and was generally lower under shading (Figure 4a). There was no significant species by radiation interaction on *R*_d_ (*P* = 0.80; Table 2). The LCP was lower under shading (Figure 4b). There was no overall significant species by radiation interaction for this trait (*P* = 0.70), and LCP was 5–7 μmol mol^−1^ for all species in dense canopy plots.

**Figure 5.**
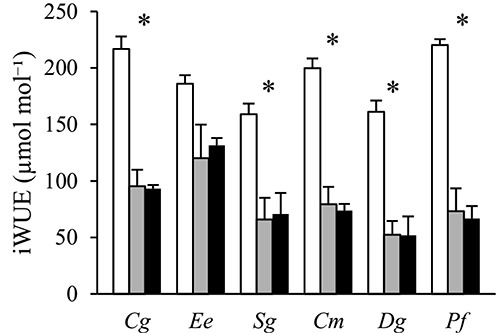
The intrinsic water-use efficiency (iWUE) determined based on leaf stable carbon isotope data from six tropical tree species grown in open (white), sparse canopy (gray) and dense canopy (black) plots. Species to the left (*Cg*, *Ee*, *Sg*) are shade-tolerant and species to the right (*Cm*, *Dg*, *Pf*) shade-intolerant. The error bars represent standard errors (*n* = 3). The symbol * indicates significant variation among radiation regimes within a species. Species: *Carapa grandiflora* (*Cg*), *Entandrophragma excelsum* (*Ee*), *Syzygium guineense* (*Sg*), *Croton megalocarpus* (*Cm*), *Dombeya goetzenii* (*Dg*) and *Polyscias fulva* (*Pf*).

Intrinsic WUE was considerably higher in open plots, and in dense canopy plots it was highest in the ST species *Cg* and *Ee* (Figure 5). There was a significant species by radiation interaction, reflecting a smaller shade-induced reduction in iWUE in ST compared with SI species (*P* = 0.015; Table 2).

### Leaf temperature

**Figure 6.**
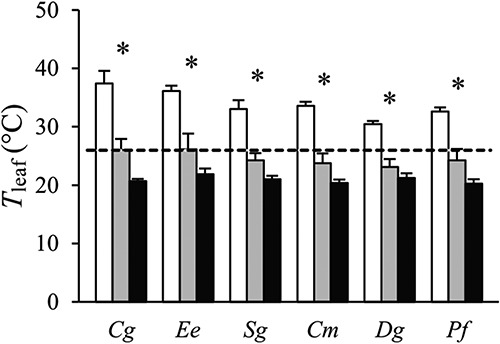
Leaf temperature (*T*_leaf_) of six tropical tree species grown in open (white), sparse canopy (gray) and dense canopy (black) plots. The dashed line indicates the Arboretum weather station air temperature (25.9 °C) during the period of the leaf temperature measurements. Species to the left (*Cg*, *Ee*, *Sg*) are shade-tolerant and species to the right (*Cm*, *Dg*, *Pf*) shade-intolerant. The error bars represent standard errors (*n* = 3). The symbol * indicates significant variation among radiation regimes within a species. Species: *Carapa grandiflora* (*Cg*), *Entandrophragma excelsum* (*Ee*), *Syzygium guineense* (*Sg*), *Croton megalocarpus* (*Cm*), *Dombeya goetzenii* (*Dg*) and *Polyscias fulva* (*Pf*).

Plant species in open plots experienced higher leaf temperatures (*T*_leaf_) compared with their counterparts under sparse and dense canopies (Figure 6). In open plots, *T*_leaf_ was always considerably higher than the prevailing air temperature measured at the local Arboretum weather station. This exceedance was 10–12 °C in the ST species *Cg* and *Ee*, while it was 5–8 °C in the other species. In dense canopy plots, *T*_leaf_ was rather similar across species, around 5 °C below the air temperature recorded by the weather station. These low *T*_leaf_ values likely resulted from a combination of low radiation, lower daytime air temperature under dense canopies compared with open plots (Table 1) and transpiratory cooling.

The species with the highest *T*_leaf_ in open plots, *Cg* and *Ee*, also had the largest leaf sizes (60–64 cm^2^ compared with 10–42 cm^2^ for the other species, Table S2 available as Supplementary Data at *Tree Physiology* Online) and the lowest stomatal conductance (Figure 3e). Their large air temperature exceedances can thus be explained by a combination of poor heat dissipation (i.e., low leaf boundary layer conductance) and low transpiratory cooling.

## Discussion

The overall aim of this study—to explore which traits were most important in controlling species shade tolerance in tropical montane trees—could be fulfilled by taking a controlled experimental approach in which we investigated a broad range of structural, chemical and physiological traits in species with different successional strategies grown under three different radiation regimes. The dense canopy plots transmitted more radiation (Table 1; 10% at noon, corresponding to about 7–8% for the daylight period if assuming 75% direct radiation and 25% diffuse radiation and spherical leaf angle distribution) than multi-layered canopies of many lowland rainforests (1–2%, Montgomery and Chazdon 2001). However, this radiation regime is representative for many late-successional stands of the highly topographical and steeply sloped montane forest from which our species originate and obviously dense enough to induce strong structural (Figure 1) and physiological (Figures 3–5) responses to shading. Our results suggest that interspecific variation in shade tolerance of tropical montane trees is controlled by species differences in whole-plant biomass allocation strategy (Figure 1) rather than by difference in physiological leaf traits determining leaf carbon balance at low radiation (Figure 4). In the shade, two of the ST species (*Cg* and *Ee*) had high fractional biomass investments in laterally light-intercepting organs (i.e., leaves, petioles and branches) and comparatively small RGR reductions below dense canopies (Figure 1). At the other end of the shade tolerance spectrum, the SI species (*Cm*, *Dg* and *Pf*) had high fractional investments in stems and larger growth reductions when shaded. The sixth species (*Sg*), which is most common in late-successional stands but also abundant in early-successional stands in Nyungwe (Nyirambangutse et al. 2017), was intermediate with respect to both RGR reductions and biomass allocation. Species with contrasting shade tolerance did not differ in leaf physiological traits controlling leaf carbon balance at low radiation (i.e., *R*_d_ and LCP; Figure 4).

### Growth and biomass allocation

Biomass allocation results were in line with our first hypothesis and provided partial support for both the carbon gain and stress tolerance hypotheses. The ST species invested relatively more into plant organs maximizing light interception (i.e., leaves and branches) than SI species (Figure 1c). In the two typical ST species (*Cg* and *Ee*), this difference was strongest under low radiation, showing that it is the result of both species adaptations and acclimation responses. The allocation strategy of these two ST species was successful with respect to total growth, as judged by the smaller growth declines under low radiation in ST compared with SI species (Figure 1a and b). Leaf morphology, however, did not vary in agreement with the carbon gain hypothesis since ST species had higher LMA than SI species in the shade (Figure 2a), in line with several previous studies on tropical tree species (Kitajima 1994, Coste et al. 2005, Mao et al. 2014). A successful ST species thus seems to need both a whole-plant architecture that is favorable for light interception (in agreement with the carbon gain hypothesis) and leaves that are strong enough to endure biotic and abiotic stress in the understorey (in agreement with the stress tolerance hypothesis; Valladares and Niinemets 2008, Valladares et al. 2016). Even though high LMA does not maximize short-term light interception and carbon gain, it might increase the carbon gain over the entire leaf lifespan thanks to better physical protection against herbivores and mechanical stresses (Kitajima and Poorter 2010, Mao et al. 2014) and longer leaf lifespan (Coste et al. 2011, Gommers et al. 2013, Reich 2014).

Shade-intolerant species grown under dense canopies had high investments in stem biomass and exhibited low RGR (Figure 1). This represents a strategy to try to escape from the shade of neighbors by rapid vertical growth (Montgomery 2004, Grubb 2015, Poorter et al. 2018). However, unless stem elongation results in increased light interception this strategy will not be successful in the long run (Henry and Aarssen 1997, Valladares et al. 2016). In this 1-year study, SI seedlings prioritizing stem growth were not rewarded by reaching higher radiation since overstorey canopies were several meters above. There was an indication that the shade-intolerant growth strategy was linked to increased mortality since seedlings that died were predominantly SI species growing below dense canopies (Figure S1 available as Supplementary Data at *Tree Physiology* Online). However, differences in mortality rates between species or radiation regimes were not statistically significant and the total number of trees that died during the experiment was rather low (21 out of 324 trees).

### Leaf physiology

Contrary to our second hypothesis, seedlings of ST and SI species did not significantly differ in *R*_d_ or LCP, two key physiological traits controlling leaf carbon balance at low radiation (Figure 4). However, light-saturated *A*_n_, photosynthetic capacity (i.e., *V*_cmax_ and *J*_max_) and stomatal conductance were generally lower in ST compared with SI species grown in open plots (Figure 3). These results indicate that light-use efficiency under low radiation is not higher in ST compared with SI species while carbon gain in open plots is clearly superior in SI species. The higher leaf-level iWUE in the ST species *Cg* and *Ee* in dense canopy plots (Figure 5) is not necessarily indicating better drought tolerance since these species also had relatively more transpiring leaf biomass and less root biomass compared with the other species (Figure 1c).

Both *R*_d_ and LCP were markedly lower in dense canopy plots, demonstrating typical sun-shade acclimation of leaf physiology (Figure 4). However, neither the degree of radiation acclimation nor the magnitude of *R*_d_ or LCP in dense canopy plots differed among species, indicating that these traits are poor predictors of species shade tolerance in tropical montane forests. This study therefore gives little support to the carbon gain hypothesis at the leaf physiological level. A previous study with tropical seedlings indicated leaf *R*_d_ to be the best predictor of shade tolerance–better than photosynthetic capacity or leaf N content (Baltzer and Thomas 2007*a*). However, that study as well as many previous investigations (Valladares et al. 2008, Coste et al. 2010, Cheng et al. 2013, Grubb 2015) have measured leaf gas exchange of understorey plants where they naturally grow, with the obvious risk of cofounding effects of acclimation and adaptation. The design of the present study allowed for the separation of acclimation and adaptation. Additional experimental studies are needed to determine if our results are valid across a broader range of species and conditions.

For *R*_d_ and LCP, acclimation to different radiation regimes was similar in ST and SI species (Figure 4) while the responses of photosynthetic capacity (i.e., *V*_cmax_ and *J*_max_) to growth radiation differed markedly between the two groups (Figure 3b and c). These findings thus only partially support previous studies on tropical trees which have indicated that sun–shade leaf plasticity is similar in ST and SI species (Rozendaal et al. 2006, Coste et al. 2009, 2010, Dusenge et al. 2015). They are rather in line with another study showing that ST species are more plastic in some traits while SI species are more plastic in others (Houter and Pons 2014). Our results indicate that interspecific variation in sun–shade leaf plasticity may markedly differ among physiological traits, but that shade tolerance is not the consequence of higher acclimation capacity of traits linked to leaf carbon balance at low radiation (i.e., *R*_d_ and LCP).

Photosynthetic capacity was lower in ST compared with SI species in open plots (Figure 3) and SPAD values (a proxy for chlorophyll content; Figure 2c) higher in ST species generally. This reflects a shift from investments in structures and compounds maximizing photosynthetic capacity towards higher investments for light harvesting in ST compared with SI species (Anderson et al. 1995, Dusenge et al. 2015). However, the link between area-based leaf N content and photosynthetic capacity appears weak since the latter but not the former differed markedly between ST and SI species (Figures 2 and 3). This result adds to a growing number of studies on tropical tree species challenging the view that leaf nutrient content is a good predictor of interspecific variation in photosynthesis (Coste et al. 2005, van de Weg et al. 2012, Houter and Pons 2014, Dusenge et al. 2015, Bahar et al. 2016, Hasper et al. 2017).

### Leaf temperature

The leaf temperatures under sunny conditions in open plots were highest in the ST species *Cg* and *Ee*, in line with our third hypothesis (Figure 6). In these species and under these conditions, leaf temperatures were on average 36–38 °C. These values clearly exceed the biochemical optimal *T*_leaf_ for light-saturated *A*_n_ (at a common intercellular CO_2_ concentration of 272 μmol mol^−1^) of these species, which are at 25–30 °C (Vårhammar et al. 2015). There were indeed indications that ST trees growing in open plots suffered in our experiment. The ST species *Cg* and *Ee* had markedly lower values of *V*_cmax_ and *J*_max_ than SI species in open plots but not in lower radiation regimes (Figure 3), which may indicate negative heat effects on photosynthetic enzymes and the electron transport chain (Sage and Kubien 2007).

It should be noted, however, that the standardized measurement of *T*_leaf_ (around noon in leaves held in horizontal position) may have caused some overestimation for leaves with steep leaf angles in open plots. The *T*_leaf_ of a horizontal leaf at noon may be comparable to that of a sun-facing leaf at 45° at 15:00h, since 14:00–15:00h is the hottest hour of the day at the Arboretum and solar radiation is then still high at an angle perpendicular to the sun. However, *Cg* in open plots had a mean leaf angle of 66° and the *T*_leaf_ measured for this species in open plots is thus an overestimation for most leaves at their natural angles (but may occur in some individual leaves with angles at or below 45 or even 30°, which also occurred).

The magnitude of leaf to air temperatures exceedance among species was linked to interspecific variation in leaf size and stomatal conductance, as also found in a previous common garden experiment with tropical ST tree seedlings (Vårhammar et al. 2015). Species with large leaf size and low stomatal conductance have poor heat dissipation and low transpiratory cooling capacity, leading to high leaf temperatures. Large leaf size and low stomatal conductance are common traits of ST species (Valladares and Niinemets 2008) and may offer partial explanation of why ST species are more negatively affected by warming than SI species in controlled experiments (Doughty 2011, Cheesman and Winter 2013b).

These results are in agreement with the emerging poly-tolerance concept which suggests that shade tolerance should be evaluated together with plant tolerance to other stressors, such as drought and waterlogging (Laanisto and Niinemets 2015, Grubb 2015, Kunstler et al. 2016, Valladares et al. 2016). Our study indicates that species growing in the understorey are not only shade-tolerant, but may in fact also be sun-intolerant at the seedling stage due to negative effects of high leaf temperature on stomatal conductance and photosynthetic biochemistry.

## Conclusions

Results from this study suggest that the large interspecific variation in shade tolerance among tropical montane trees is controlled by whole-plant biomass allocation strategy rather than by variation in physiological leaf traits determining leaf carbon balance at low radiation. Species with varying shade tolerance had distinctly different biomass allocation patterns (Figure 1): the two distinct ST species (*Cg* and *Ee*) invested relatively more into plant organs maximizing light interception in the understorey (i.e., leaves and branches) while SI species invested more into stems. These differences in fractional biomass investments were more pronounced under dense canopies. This shows that ST and SI species in our tropical montane forest have two fundamentally different strategies to deal with low-light conditions: ST species maximize light-use efficiency in the shade while SI species try to escape the deep shade through vertical stem growth. Contrary to our expectations, however, ST and SI species did not differ in leaf *R*_d_ or LCP, i.e., traits controlling leaf carbon balance at low radiation (Figure 4).

Our results highlight the importance of a whole-plant perspective for understanding tree shade tolerance. Based on leaf level physiological data only, we found little support for the carbon gain hypothesis (stating that shade tolerance is the consequence of high light use-efficiency resulting from traits maximizing carbon gain and minimizing carbon losses in a low-light environment). However, based on both leaf physiological and whole-plant structural data we can conclude that the carbon gain hypothesis is relevant, but that carbon gain of our two distinct ST species is increased by biomass allocation strategy rather than by leaf level physiological traits.

## Supplementary Material

Ntawuhiganayo_supplementary_Re-re-revision_tpz119
