# Supplementary material for: Traits controlling shade tolerance in tropical montane trees
Source: Tree Physiol. 2019 Dec 19;40(2):183–97. doi: 10.1093/treephys/tpz119 (PMC7048680; doi:10.1093/treephys/tpz119)
Supplement: Ntawuhiganayo_supplementary_Re-re-revision_tpz119 [file ntawuhiganayo_supplementary_re-re-revision_tpz119.pdf]

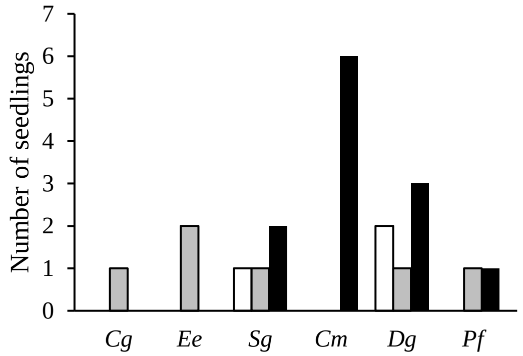

Figure S1. The tree mortality (number of individual seedlings) of *Carapa grandiflora* (*Cg*), *Entandrophragma excelsum* (*Ee*), *Syzygium guineense* (*Sg*), *Croton megalocarpus* (*Cm*), *Dombeya goetzenii* (*Dg*) and *Polyscias fulva* (*Pf*) planted in open-canopy (white), sparse-canopy (gray) and dense-canopy (black).

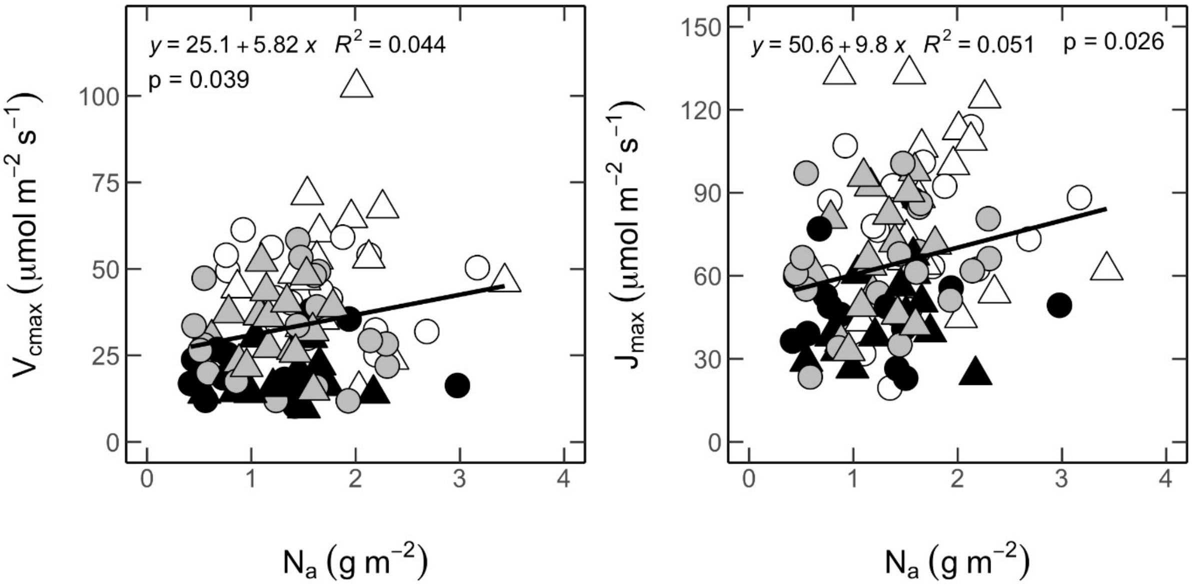

Figure S2. The (**a**) the maximum rates of photosynthetic carboxylation (V_{cmax}) and (**b**) electron transport (J_{max}) in relation to leaf nitrogen content per unit leaf area (N_a). Each data point represents an individual leaf of shade tolerant (triangles) and shade intolerant (circle) tree species grown in open (white), sparse canopy (gray) and dense canopy (black) plots. The dark lines represent the linear regression for V_{cmax} ($y = 5.87x + 24.6$) and J_{max} ($y = 9.8x + 50.6$).

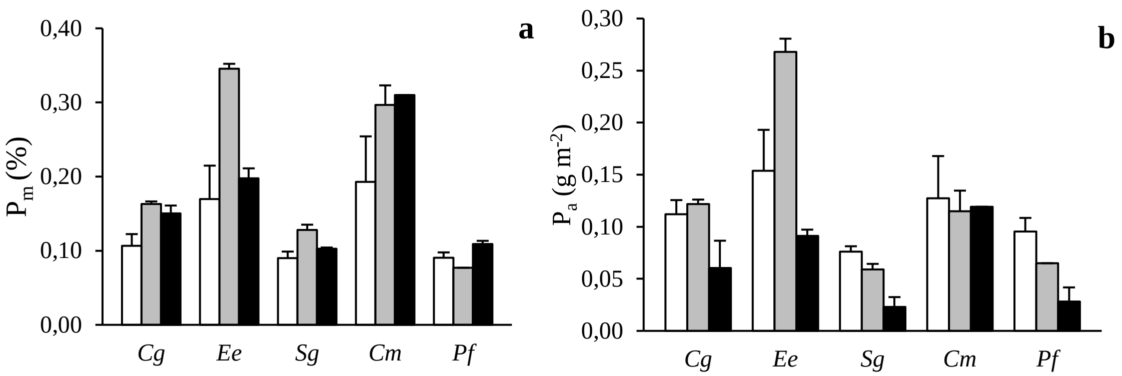

Figure S3. Leaf **(a)** mass-based (Pm) and **(b)** area-based phosphorus (Pa) content of six tropical tree species grown in open (white), sparse canopy (gray) and dense canopy (black) plots. The error bars represent standard errors (*n* = 3). Species: *Carapa grandiflora* (*Cg*), *Entandrophragma excelsum* (*Ee*), *Syzygium guineense* (*Sg*), *Croton megalocarpus* (*Cm*) and *Polyscias fulva* (*Pf*).

Table S1. Initial harvest data LMA and the biomass of roots, stems, and branches with leaves for *Carapa grandiflora* (*Cg*), *Entandrophragma excelsum* (*Ee*), *Syzygium guineense* (*Sg*), *Croton megalocarpus* (*Cm*), *Dombeya goetzenii* (*Dg*) and *Polyscias fulva* (*Pf*). Values are mean ± SE.

	**Roots (g)**	**Stem (g)**	**Branches + leaves (g)**	**Total mass (g)**	**LMA (g m^{-2})**
Cg	2.95 ± 0.26	3.40 ± 0.28	2.13 ± 0.23	8.22 ± 0.67	68.2 ± 1.1
Ee	2.17 ± 0.23	3.43 ± 0.37	3.85 ± 0.56	9.45 ± 1.11	62.8 ± 3.5
Sg	1.38 ± 0.13	1.79 ± 0.23	1.61 ± 0.12	4.78 ± 0.46	89.8 ± 3.0
Cm	3.10 ± 0.55	8.46 ± 1.21	2.82 ± 0.74	14.38 ± 2.22	40.5 ± 2.3
Dg	1.77 ± 0.18	4.75 ± 0.36	1.08 ± 0.13	7.60 ± 0.50	42.8 ± 6.4
Pf	1.48 ± 0.20	3.52 ± 0.44	1.83 ± 0.84	6.84 ± 0.85	53.8 ± 8.7

Table S2. Entire database, including physiological, chemical and structural leaf traits as well as tree biomass and its allocation. Values are mean ± SE.

		Plant species						
**Leaf traits**		***C. grandiflora***	***E. excelsum***	***S. guineense***	***C. megalocarpus***	***D. goetzenii***	***P. fulva***	***All***
	**OP**	30.5 ± 1.2	30.5 ± 5.0	45.2 ± 1.8	51.6 ± 5.5	62.8 ± 8.5	48.9 ± 5.1	44.9 ± 1.8
V_{cmax}	**SP**	41.1 ± 5.0	27.5 ± 3.6	22.8 ± 3.4	31.6 ± 6.3	36.2 ± 3.3	30.7 ± 3.4	31.7 ± 3.0
(µmol $m^{-2}s^{-1}$)	**DP**	34.2 ± 1.6	19.0 ± 1.6	14.9 ± 2.1	17.9 ± 0.4	20.9 ± 1.7	17.5 ± 1.1	21.0 ± 0.3
	**OP**	53.1 ± 5.7	55.2 ± 3.3	88.5 ± 7.9	84.8 ± 13.5	107.6 ± 5.1	75.2 ± 9.1	77.4 ± 2.5
J_{max}	**SP**	65.1 ± 7.5	70.2 ± 10.7	63.6 ± 4.4	62.3 ± 8.6	69.3 ± 5.3	62.9 ± 10.9	65.6 ± 1.9
(µmol $m^{-2}s^{-1}$)	**DP**	52.9 ± 15.3	47.0 ± 7.0	44.3 ± 2.8	48.6 ± 1.7	45.7 ± 6.5	46.3 ± 6.8	48.1 ± 4.7
	**OP**	1.62 ± 0.05	1.96 ± 0.27	1.99 ± 0.09	1.65 ± 0.07	1.84 ± 0.17	1.54 ± 0.12	1.77 ± 0.07
J_{max}/V_{cmax}	**SP**	1.87 ± 0.43	2.60 ± 0.19	3.13 ± 0.31	2.22 ± 0.53	1.89 ± 0.09	2.02 ± 0.18	2.29 ± 0.22
	**DP**	1.49 ± 0.37	2.52 ± 0.26	3.29 ± 0.61	2.71 ± 0.07	2.15 ± 0.15	2.64 ± 0.25	2.48 ± 0.23
	**OP**	0.70 ± 0.04	0.87 ± 0.07	0.78 ± 0.11	0.67 ± 0.08	0.76 ± 0.16	0.61 ± 0.10	0.73 ± 0.07
R_d	**SP**	0.72 ± 0.15	0.50 ± 0.16	0.63 ± 0.11	0.54 ± 0.14	0.65 ± 0.12	0.58 ± 0.15	0.60 ± 0.10
(µmol $m^{-2}s^{-1}$)	**DP**	0.52 ± 0.08	0.35 ± 0.06	0.59 ± 0.16	0.37 ± 0.04	0.37 ± 0.02	0.38 ± 0.02	0.43 ± 0.04
	**OP**	12.9 ± 0.3	13.4 ± 0.8	11.1 ± 1.9	11.4 ± 0.9	13.0 ± 2.0	9.6 ± 1.5	12.9 ± 0.3
**LCP**	**SP**	10.6 ± 0.8	6.9 ± 1.9	9.7 ± 0.9	7.0 ± 1.6	8.5 ± 2.4	8.2 ± 2.9	10.6 ± 0.8
(µmol mol^{-1})	**DP**	6.3 ± 0.6	5.1 ± 0.5	6.7 ± 0.4	6.2 ± 0.5	5.6 ± 2.7	5.8 ± 0.5	6.3 ± 0.6
	**OP**	0.03 ± 0.01	0.05 ± 0.01	0.14 ± 0.01	0.16 ± 0.05	0.26 ± 0.08	0.15 ± 0.05	0.13 ± 0.03
***G*s**	**SP**	0.04 ± 0.00	0.03 ± 0.01	0.07 ± 0.02	0.08 ± 0.01	0.15 ± 0.06	0.11 ± 0.03	0.08 ± 0.02
(mol H_2O $m^{-2}s^{-1}$)	**DP**	0.04 ± 0.01	0.02 ± 0.00	0.03 ± 0.00	0.07 ± 0.02	0.05 ± 0.01	0.12 ± 0.05	0.05 ± 0.01
	**OP**	1.80 ± 0.64	1.57 ± 0.65	6.68 ± 0.30	6.83 ± 1.33	8.22 ± 1.80	5.62 ± 1.57	5.12 ± 0.89
A_n	**SP**	2.78 ± 0.41	2.56 ± 0.35	3.30 ± 0.81	4.51 ± 0.55	4.98 ± 1.11	3.67 ± 0.35	3.63 ± 0.42
(µmol $m^{-2}s^{-1}$)	**DP**	2.88 ± 0.98	2.11 ± 0.40	1.46 ± 0.09	2.65 ± 0.48	2.88 ± 0.51	2.26 ± 0.45	2.43 ± 0.43
	**OP**	0.11 ± 0.01	0.15 ± 0.04	0.08 ± 0.01	0.15 ± 0.04	-	0.06 ± 0.01	0.10 ± 0.01
P_a	**SP**	0.08 ± 0.00	0.27 ± 0.01	0.06 ± 0.01	0.11 ± 0.02	-	0.02 ± 0.00	0.08 ± 0.02
(g m^{-2})	**DP**	0.06 ± 0.03	0.09 ± 0.01	0.02 ± 0.01	0.04 ± 0.00	-	0.02 ± 0.01	0.04 ± 0.00

	**OP**	0.10 ± 0.02	0.17 ± 0.05	0.09 ± 0.01	0.23 ± 0.06	-	0.06 ± 0.01	0.12 ± 0.02
**P_m**	**SP**	0.11 ± 0.00	0.35 ± 0.01	0.13 ± 0.01	0.30 ± 0.03	-	0.03 ± 0.00	0.13 ± 0.03
(%)	**DP**	0.15 ± 0.01	0.20 ± 0.01	0.10 ± 0.00	0.10 ± 0.00	-	0.07 ± 0.00	0.10 ± 0.02
	**OP**	2.32 ± 0.11	1.55 ± 0.13	1.16 ± 0.10	1.66 ± 0.07	1.91 ± 0.07	1.75 ± 0.18	1.72 ± 0.02
**N_a**	**SP**	2.01 ± 0.12	1.47 ± 0.06	0.63 ± 0.07	1.18 ± 0.05	1.00 ± 0.10	1.52 ± 0.11	1.30 ± 0.01
(g m^{-2})	**DP**	1.88 ± 0.20	1.35 ± 0.18	0.53 ± 0.01	1.35 ± 0.12	1.25 ± 0.04	1.63 ± 0.03	1.32 ± 0.04
	**OP**	1.74 ± 0.05	1.65 ± 0.06	1.34 ± 0.05	2.59 ± 0.14	3.68 ± 0.46	1.65 ± 0.15	2.11 ± 0.07
**N_m**	**SP**	2.02 ± 0.13	1.96 ± 0.12	1.37 ± 0.07	2.73 ± 0.23	3.39 ± 0.21	2.16 ± 0.15	2.27 ± 0.13
(%)	**DP**	2.40 ± 0.04	2.82 ± 0.10	1.60 ± 0.02	3.87 ± 0.39	4.48 ± 0.23	3.06 ± 0.17	3.04 ± 0.14

Leaf traits		Plant species *C. grandiflora*	*E. excelsum*	*S. guineense*	*C. megalocarpus*	*D. goetzenii*	*P. fulva*	*All*
	**OP**	88.3 ± 4.2	98.9 ± 5.5	185.5 ± 5.7	70.1 ± 5.6	47.2 ± 3.2	99.2 ± 3.4	98.2 ± 3.7
**LMA**	**SP**	67.6 ± 5.3	65.2 ± 6.4	122.8 ± 22.9	43.8 ± 6.6	30.4 ± 6.1	51.1 ± 11.7	63.5 ± 9.8
(g m^{-2})	**DP**	56.3 ± 1.8	48.8 ± 1.1	99.3 ± 16.8	28.9 ± 4.6	17.8 ± 0.7	28.6 ± 2.9	47.1 ± 4.5
	**OP**	16.3 ± 0.3	16.4 ± 1.7	11.4 ± 0.4	11.4 ± 0.6	9.3 ± 0.3	9.6 ± 0.6	12.4 ± 0.4
**Leaf length**	**SP**	17.9 ± 0.4	21.4 ± 1.1	12.7 ± 0.3	13.6 ± 0.7	13.6 ± 0.8	11.0 ± 1.1	15.0 ± 0.6
(cm)	**DP**	17.7 ± 0.5	21.7 ± 0.9	13.8 ± 0.5	12.7 ± 1.8	11.7 ± 0.7	13.1 ± 0.6	15.3 ± 0.6
	**OP**	6.23 ± 0.33	7.14 ± 0.47	3.12 ± 0.23	5.90 ± 0.31	8.39 ± 0.47	4.31 ± 0.24	5.85 ± 1.10
**Leaf width**	**SP**	5.91 ± 0.13	8.69 ± 0.57	3.84 ± 0.11	6.69 ± 0.04	12.92 ± 0.65	5.04 ± 0.38	7.18 ± 0.23
(cm)	**DP**	6.54 ± 0.06	8.51 ± 0.44	4.13 ± 0.48	8.09 ± 0.80	10.12 ± 0.20	5.45 ± 0.26	7.28 ± 0.28
	**OP**	0.25 ± 0.01	0.27 ± 0.00	0.29 ± 0.02	0.26 ± 0.01	0.25 ± 0.01	0.29 ± 0.01	0.27 ± 0.01
**Leaf thickness**	**SP**	0.21 ± 0.01	0.21 ± 0.01	0.27 ± 0.02	0.20 ± 0.01	0.19 ± 0.01	0.24 ± 0.02	0.22 ± 0.01
(mm)	**DP**	0.17 ± 0.01	0.18 ± 0.00	0.21 ± 0.01	0.16 ± 0.00	0.18 ± 0.01	0.19 ± 0.02	0.18 ± 0.01
	**OP**	64.3 ± 3.5	60.1 ± 1.0	10.5 ± 1.3	22.9 ± 4.2	42.3 ± 21.6	18.0 ± 0.7	36.4 ± 4.6
**Leaf size**	**SP**	71.2 ± 7.2	135.7 ± 16.3	16.5 ± 2.6	48.4 ± 9.4	104.3 ± 22.9	31.2 ± 6.4	67.9 ± 10.6
(cm^2)	**DP**	67.5 ± 3.2	110.1 ± 1.6	16.6 ± 3.8	55.8 ± 4.9	40.3 ± 4.9	26.8 ± 4.4	52.0 ± 2.0
	**OP**	66 ± 2	27 ± 2	44 ± 3	52 ± 2	34 ± 5	56 ± 3	47 ± 1
**Leaf angle**	**SP**	33 ± 2	25 ± 2	29 ± 2	30 ± 5	20 ± 2	20 ± 7	26 ± 3
(^{o})	**DP**	24 ± 1	16 ± 1	16 ± 1	25 ± 3	17 ± 3	14 ± 2	19 ± 1
	**OP**	51.2 ± 0.8	44.3 ± 4.4	52.0 ± 1.5	34.3 ± 1.8	31.0 ± 1.0	32.9 ± 1.1	41.0 ± 0.9
**SPAD**	**SP**	60.3 ± 2.5	54.9 ± 4.9	50.5 ± 2.9	43.6 ± 3.6	33.6 ± 2.0	31.9 ± 1.3	45.8 ± 2.6
	**DP**	69.6 ± 0.6	64.8 ± 2.4	57.9 ± 1.5	44.3 ± 1.6	34.3 ± 3.9	31.9 ± 0.4	50.6 ± 0.9

Tree traits		Plant species *C. grandiflora*	*E. excelsum*	*S. guineense*	*C. megalocarpus*	*D. goetzenii*	*P. fulva*	*All*
**RGR**	**OP**	2.31 ± 0.07	2.32 ± 0.04	3.09 ± 0.15	1.72 ± 0.02	2.39 ± 0.17	2.79 ± 0.07	2.35 ± 0.07
	**SP**	2.23 ± 0.11	2.45 ± 0.12	2.54 ± 0.11	1.43 ± 0.11	2.29 ± 0.11	2.17 ± 0.02	2.17 ± 0.04
	**DP**	1.25 ± 0.05	1.53 ± 0.12	1.59 ± 0.06	0.08 ± 0.28	1.15 ± 0.12	1.13 ± 0.14	1.18 ± 0.04
**Roots Weight** (g)	**OP**	35.6 ± 3.2	26.8 ± 1.2	41.9 ± 5.6	34.5 ± 1.2	41.1 ± 7.4	30.2 ± 2.7	33.7 ± 1.5
	**SP**	26.7 ± 4.7	29.9 ± 3.7	18.7 ± 1.5	18.0 ± 2.2	26.6 ± 5.1	17.3 ± 0.8	23.0 ± 2.4
	**DP**	10.6 ± 0.8	12.5 ± 1.2	7.1 ± 0.3	5.1 ± 1.9	8.1 ± 0.8	5.6 ± 0.7	8.5 ± 0.5
**Stem Weight** (g)	**OP**	30.0 ± 2.1	31.9 ± 0.8	33.0 ± 6.4	30.7 ± 0.6	32.0 ± 2.7	51.9 ± 2.9	34.7 ± 1.7
	**SP**	34.3 ± 2.1	36.9 ± 3.9	27.9 ± 1.7	32.2 ± 3.4	40.2 ± 2.7	27.0 ± 1.0	33.2 ± 0.6
	**DP**	12.4 ± 0.9	14.6 ± 1.7	9.3 ± 0.3	9.6 ± 2.0	14.0 ± 2.1	11.3 ± 1.6	12.0 ± 0.8
**Branches+Petioles** (g)	**OP**	4.52 ± 0.32	3.82 ± 0.63	9.52 ± 0.40	5.59 ± 0.25	3.22 ± 0.11	4.58 ± 1.30	4.95 ± 0.36
	**SP**	6.48 ± 0.33	6.45 ± 0.66	5.94 ± 0.80	3.38 ± 0.55	2.52 ± 1.09	4.25 ± 0.78	4.82 ± 0.38
	**DP**	2.79 ± 0.01	2.85 ± 0.44	1.51 ± 0.34	0.97 ± 0.49	1.14 ± 0.14	1.14 ± 0.43	1.85 ± 0.21
**Leaves Weight** (g)	**OP**	17.0 ± 1.7	14.6 ± 2.3	21.2 ± 3.0	6.5 ± 0.6	2.6 ± 0.9	12.3 ± 1.5	12.3 ± 1.3
	**SP**	23.3 ± 1.4	21.8 ± 2.7	16.9 ± 1.1	7.9 ± 2.1	3.8 ± 0.8	8.7 ± 1.4	13.8 ± 1.5
	**DP**	10.7 ± 0.3	11.1 ± 1.2	4.9 ± 1.0	2.6 ± 1.4	1.1 ± 0.4	1.7 ± 0.6	5.9 ± 0.3
**Total Weight** (g)	**OP**	87.5 ± 5.0	77.5 ± 4.5	106.0 ± 15.5	77.6 ± 1.3	78.9 ± 10.0	99.2 ± 5.4	86.0 ± 3.0
	**SP**	91.1 ± 5.0	95.3 ± 8.3	69.8 ± 2.1	61.7 ± 4.5	73.2 ± 8.7	57.4 ± 1.3	75.0 ± 2.3
	**DP**	36.8 ± 1.5	41.3 ± 4.5	23.0 ± 1.7	18.4 ± 5.8	24.4 ± 3.4	19.8 ± 3.3	28.4 ± 1.8
**Root/Shoot ratio**	**OP**	0.75 ± 0.10	0.55 ± 0.02	0.68 ± 0.01	0.82 ± 0.02	1.12 ± 0.19	0.44 ± 0.03	0.70 ± 0.05
	**SP**	0.42 ± 0.07	0.46 ± 0.04	0.39 ± 0.05	0.43 ± 0.08	0.58 ± 0.10	0.44 ± 0.03	0.45 ± 0.05
	**DP**	0.43 ± 0.02	0.45 ± 0.01	0.44 ± 0.01	0.38 ± 0.03	0.53 ± 0.03	0.42 ± 0.02	0.44 ± 0.01
**Wood density** (g/cm^3)	**OP**	0.68 ± 0.00	0.55 ± 0.02	0.67 ± 0.03	0.68 ± 0.01	0.48 ± 0.02	0.43 ± 0.02	0.58 ± 0.01
	**SP**	0.52 ± 0.02	0.57 ± 0.01	0.68 ± 0.03	0.68 ± 0.02	0.45 ± 0.01	0.37 ± 0.01	0.54 ± 0.01
	**DP**	0.55 ± 0.01	0.54 ± 0.01	0.64 ± 0.01	0.59 ± 0.02	0.35 ± 0.02	0.37 ± 0.03	0.51 ± 0.01

	**OP**	1.35 ± 0.03	1.56 ± 0.03	1.13 ± 0.06	1.24 ± 0.04	1.29 ± 0.02	1.83 ± 0.06	1.40 ± 0.02
**Base diameter**	**SP**	1.42 ± 0.06	1.70 ± 0.10	1.19 ± 0.03	1.14 ± 0.04	1.38 ± 0.03	1.52 ± 0.04	1.39 ± 0.03
(cm)	**DP**	0.90 ± 0.03	1.14 ± 0.04	0.75 ± 0.03	0.63 ± 0.08	0.95 ± 0.03	1.14 ± 0.06	0.91 ± 0.02
	**OP**	47.4 ± 2.1	47.8 ± 0.7	81.6 ± 4.1	89.8 ± 3.4	111.9 ± 2.0	70.5 ± 3.4	74.8 ± 1.2
**Stem height**	**SP**	87.8 ± 3.5	51.9 ± 0.9	108.7 ± 14.2	114.2 ± 7.8	164.9 ± 6.1	71.0 ± 4.0	99.8 ± 3.7
(cm)	**DP**	57.1 ± 2.2	48.7 ± 3.3	78.6 ± 3.5	91.9 ± 1.8	124.6 ± 2.5	68.2 ± 3.8	77.8 ± 1.1

V_{cmax}: maximum rates of photosynthetic carboxylation, J_{max}: maximum rates of electron transport, R_d: leaf dark respiration, **LCP**: light compensation point, g_s: stomatal conductance, A_n: light-saturated net CO_2 assimilation, P_a: area-based Phosphorus content, P_m: mass-based Phosphorus content, N_a: Nitrogen content per unit leaf area, N_m: Nitrogen content per unit leaf mass, **LMA**: leaf mass per unit leaf area, **SPAD**: proxy for leaf chlorophyll content.
